# Mycobiota contaminating some market cake samples with reference to their toxin and enzyme

**DOI:** 10.1186/s12866-024-03345-x

**Published:** 2024-06-14

**Authors:** Shimaa M. Abdelhameed, Basma A. Khalifa

**Affiliations:** Botany and Microbiology Department, Faculty of Science, Minia University, Minia city, Egypt

**Keywords:** Cake spoilage, Mycoflora, Enzymes production, Mycotoxins, Aflatoxins, Essential oils

## Abstract

Fungi can spoil the majority of baked products. Spoilage of cake during storage is commonly associated with fungi. Therefore, this study aimed to assess the quality of different types of cakes sold in the market. The most predominant fungal genera in the tested cake samples (14 samples) were *Aspergillus* spp., and *Penicillium* spp. On Potato Dextrose Agar (PDA), the medium fungal total count was 43.3 colonies /g. *Aspergillus* was the most dominant genus and was isolated from six samples of cake. *Aspergillus* was represented by 3 species namely, *A. flavus*, *A. niger,* and *A. nidulans,* represented by 13.32, 19.99, and 3.33 colonies /g respectively. On Malt Extract Agar (MEA) Medium, the fungal total count was 123.24 colonies / g*. Aspergillus* was the most dominant isolated genus from 11 samples of cake and was represented by 5 species, namely*, A. flavus*, *A. niger*, *A. ochraceous*, *A. terreus,* and *A. versicolor* (26. 65, 63.29, 3.33, 6.66, and 3.33 colonies / g , respectively). Twenty-four isolates (88.88 %) of the total tested twenty-seven filamentous fungi showed positive results for amylase production. Ten isolates (37.03%) of the total tested filamentous fungi showed positive results for lipase production, and finally eleven isolates (40.74 %) of the total fungal isolates showed positive results for protease production. Aflatoxins B_1_, B_2,_ G_1,_ G_2,_ and ochratoxin A were not detected in fourteen collected samples of cake. In this study, clove oil was the best choice overpeppermint oil and olive oil for preventing mold development when natural agents were compared. It might be due to the presence of a varietyof bioactive chemical compounds in clove oil, whose major bioactive component is eugenol, which acts as an antifungal reagent. Therefore, freshly baked cake should be consumed within afew days to avoid individuals experiencing foodborne illnesses.

## Introduction

The issue of food losses is of major relevance in the efforts to eliminate hunger, generate income, and enhance food security in the world’s poorest countries. Food losses affect the ecology, economic growth, food security for the poor, and food quality and safety. It has only become obvious for the past 30 years that mycotoxins, which are toxic substances, can be produced by common fungi that grow in foods and feeds. In past times, these toxins have led to significant epidemics in both humans and animals. Egotism, which claimed the lives of hundreds of thousands of people in Europe in the previous millennium [1], alimentary toxic leukemia, which killed at least 100,000 Russians between 1942 and 1948 [2], stachybotryotoxicosis, which asserted the lives of tens of thousands of horses in the USSR in the 1930s [3], and mycotoxicosis, which killed 100,000 young turkeys in the UK in 1960 and since has spread to other animals and humans [4].

Any sweet dessert that is commonly baked is referred to as cake [5]. In their oldest forms, cakes were a modification of bread but covered a wide range of preparations that could be simple or complicated. Typical, cake ingredients are flour, sugar, eggs, butter or oil, a liquid, in addition to leavening agents like baking soda and/or baking powder. Though molds are eliminated in the baking process, mold spores from the atmosphere or surfaces during the cooling, finishing, and packaging stages cause contamination [6]. These products typically have a water activity (aw) of 0.70 to 0.85, though occasionally they may have a greater aw if condensation forms on their surface as a result of a temperature gradient.

The appearance of fungus colonies in cake recipes might result in customer rejection and financial losses [7]. Due to their prolonged storage (> 45 days), low water activity (0.78-0.95), and preservative content, industrialized cakes are prone to fungal deterioration [7]. Fungi are to blame for the degradation of manufactured foods and raw resources, especially bakery goods. Fruits and breads are more likely to be ruined by fungus than by bacteria because fungi thrive in acidic and low moisture settings. Fungi are more frequently to blame for food spoiling than for food-borne illnesses because they have a high tolerance for acidic conditions [5]. Even though microbial contamination before baking is well-known, it is not thought to be the most important problem because baking may eliminate the mycobiota. The microbiological stability of cakes appears to be severely harmed by post-baking contamination (air, product handling, and improperly sterilized equipment) [8]. The most frequent cause of microbial spoilage is the formation of mold. In accordance with the earlier research, It has been observed that during bread spoilage, bread molds like *Mucor* and *Rhizopus* develop first. This is followed by some fungi, such as *Aspergillus*, *Penicillium*, and *Fusarium* sp. [9].

The Food and Agriculture Organization (FAO) estimates that mycotoxins produced by storage molds globally cause the spoilage of around 1000 million metric tons of food goods each year [10]. For both people and animals, mycotoxins and other secondary metabolites pose serious health risks [11]. Food contamination from various toxigenic molds (fungi) is a serious but mostly ignored problem. It can result in large economic losses, and their presence in food has an effect on its quality. According to Moosavi et al. [12], mycotoxins and other secondary metabolites are produced by filamentous fungi *Fusarium*, *Aspergillus* and *Penicillium* genera. Mycotoxins are hazardous compounds that may appear on a variety of crops and food products, including baked goods. They are created by certain molds and fungi. When ingested in even small quantities, these hazardous compounds pose serious health concerns to both humans and animals. During the process of cultivation, harvest, transportation, and storage of raw ingredients used in bakery products, mycotoxins can contaminate them and pose a risk to public health. Molds like *Aspergillus*, *Penicillium*, and *Fusarium* species produce mycotoxins. These molds flourish best in warm, humid environments with improper storage techniques. A few examples of the variables that affect mycotoxin formation are temperature, moisture content, and light exposure. Regarding bakery goods, there are a few mycotoxins that should be especially avoided: Aflatoxin; produced by *Aspergillus* species is the main source of these extremely potent carcinogens. They also possess the potential to contaminate raw components used in baking goods, such as grains and nuts. Ochratoxin A: This mycotoxin, which is produced by several *Penicillium* and *Aspergillus* species, is present in cereal-based foodstuffs like bread and is associated with kidney damage. There are four main categories of mycotoxin toxicity: mutagenic, teratogenic, chronic, and acute. Acute mycotoxin poisoning is most usually associated with liver or renal dysfunction, which can be fatal in severe circumstances. Nonetheless, certain mycotoxins mainly function by disrupting the synthesis of proteins, resulting in a variety of consequences, such as severe immunodeficiency, skin sensitivity, or necrosis. Others are neurotoxins, which only slowly increase in dose to the point where they induce brain damage or even death in animals but may produce prolonged shaking in small doses. Ingesting low concentrations of mycotoxin can have a variety of long-term impacts. The primary long-term consequence of several mycotoxins is the promotion of cancer, particularly hepatic cancer. Certain toxins have mutagenic or teratogenic effects because they interfere with DNA replication [4, 13]. Acute stomach pain, vomiting, as well as chronic illnesses like cancer, immunological suppression, hepatomegaly, stunting, and ultimately death, are all possible toxicological effects [14, 15].

The extracellular enzymes amylatic, proteolytic, and lipolytic are produced by most isolated fungal species, which affect the product quality [16]. Vegetables, bread, fruits, nuts, chocolates, cereals, coffee beans, liquor, and legumes can all harbor mycotoxin-producing fungi [17]. The sensory qualities of food items are deteriorating due to mycotoxins' production of many enzymes and certain volatiles, which lowers food quality [18]. Clove essential oil is generally recognized as safe by the Food and Drug Administration. Humans can benefit from clove essential oil's antibacterial, antioxidant, and insecticidal properties [19, 20]. Chemical preservatives like formaldehyde, sodium nitrite, sulfur dioxide, propionates, and sulfites have decreased food microbial contamination, according to modern production processes. Nevertheless, this has a negative effect on environmental sustainability and serious negative health effects. Foodborne illnesses have become an important issue for the food industry, especially those caused by bacteria, fungus, and related toxins. Artificial food preservatives have the potential to harm the heart and nerves over time. EOs, in particular, are gaining popularity as plant-based preservatives. EOs are not dangerous to mammals, according to the US Food and Drug Administration. According to studies, essential oils of clove and cinnamon have a higher potential for exhibiting significant antibacterial activity against extended-spectrum beta-lactamases, producing Klebsiella, pneumonia, and Escherichia coli isolates, This confirms that the oils are potent antibacterial agents [21, 22]. A decrease in artificial food additives is being demanded by consumers due to concerns about the effects of these artificial chemical compounds on health. Therefore, one way to improve product safety and shelf life while maintaining consumer preferences could be to add antimicrobial compounds from natural sources to food [22]. Although several studies have sought to clarify the reason for the microbial deterioration of bakery goods [23], little is known about their microbial biodiversity. In this study, the frequency and abundance of fungus in the chain of cake manufacturing were examined. The objectives of this study are the isolation and identification of spoilage fungi from some market cake through morphological techniques and to check the presence of aflatoxins and ochratoxin A contamination. To determine the ability of isolates to produce amylase, protease, and lipase enzymes and to evaluate the antifungal efficacy of essential oils (clove, peppermint, and olive oil) against cake spoilage molds. To show the importance of using essential oils as natural preservatives instead of artificial preservatives.

## Materials and methods

### Collection of cake samples

A total of 14 selected samples of cake were collected from different supermarkets in Minia Governorate, and the time of sample collection was on 10 October, 2022. Samples were transferred to the laboratory and kept in a refrigerator (4°C) for fungal examination and mycotoxin analysis. The data in Table 1 indicates production and expiration dates in addition to the ingredients of cake samples.

**Table 1:** Production and Expiration date in addition to ingredients of cake samples

Sample no.	Production date	Expiration date	Ingredients
1	28-9-2022	26-1-2023	Wheat flour-eggs-dextrose powder-fructose syrup-vegetable oils-xanthan gum-stabilizers-raising materials-taste additives-natural color-table salt-food emulsifiers.
2	21-8-2022	20-2-2023	Wheat flour-sugar-palm oil-glucose syrup-eggs-milk powder-cocoa powder- moisturizing substances- food emulsifiers- raising materials-corn oil- stabilizers-preservatives-starch-table salt-soy lecithin-acidity regulators.
3	3-10-2022	2-1-2023	Wheat flour-sugar-eggs-glucose syrup-sunflower oil-vegetable oils- soy oil -milk powder- moisturizing substances- food emulsifiers- raising materials- preservatives-starch-table salt
4	17-10-2022	16-2-2023	Wheat flour-sugar-eggs-glucose syrup-sunflower oil-vegetable oils- soy oil -milk powder- moisturizing substances- food emulsifiers-raising materials- preservatives-starch-table salt-nut butter-acidity regulators.
5	4-7-2022	3-1-2023	Wheat flour-sugar-eggs-non hydrogenated vegetable oil(palm)- dried fruits 7 %(papaya or mango or pinapple or cantaloupe or raisin sugar syrup-acidity regulator (citric acid)-permitted food colors-water-glucose syrup-humectant(sorbitol gum)-corn starch-raising agents-(sodium acid pyrophosphate-sodium bicarbonate)-vegetable edible emulsifiers-propylene glycerol ester of fatty acids-whey powder-salt-beta carotene-identical natural lemon flavor-preservative with permitted ratios(potassium sorbate)-thickener(xanthan gum)-vanillin-all contents allowed limits-registered trademark.
6	3-10-2022	2-1-2023	Wheat flour-sugar-palm oil-cocoa butter substitute-glucose syrup-fructose syrup-cocoa powder-whey milk-food glycerin-propylene glycol-preservatives(sorbic acid and salts)- food emulsifiers-soy flour- raising materials- food emulsifiers- taste additives-table salt-vanillin-citric acid-xanthan gum-sorbitol-CMC-cottonseed oil-sodium citrate-sodium benzoate-sodium acetate.
7	7-8-2022	6-1-2023	Wheat flour-eggs-dextrose powder-fructose syrup-vegetable oils-xanthan gum-stabilizers-raising materials-taste additives-natural color-table salt-food emulsifiers.
8	28-9-2022	26-1-2023	Wheat flour-eggs-dextrose powder-fructose syrup-vegetable oils-xanthan gum-stabilizers-raising materials-taste additives (strawberry)-natural color-table salt-food emulsifiers.
9	6-10-2022	5-2-2023	Gluten-eggs-soya and milk-May contain traces of nuts and derivatives.
10	7-10-2022	6-2-2023	Wheat flour-sugar-eggs-vegetable oils-cow butter-moisturizer-milk powder-raising materials-starch-preservatives-soy proteins-xanthan gum-flavors similar to nature.
11	7-9-2022	6-3-2023	Wheat flour-sugar-hydrogenated vegetable oils-dextrose-fructose syrup-cocoa powder-milk powder- table salt-raising materials-preservatives-xanthan gum-moisturizing substances- food emulsifiers.
12	7-9-2022	6-6-2023	Hydrogenated and non-hydrogenated vegetable oils-sugar-eggs-cocoa powder-glucose syrup-fructose syrup-raw cocoa-emulsifiers- moisturizer-wheat starch-dextrose-milk powder-raising materials- whey-skimmed milk powder-modified starch-antimicrobial preservatives and antioxidants- flavors similar to nature (vanillin-caramel-milk-chocolate)-natural color.
13	8-8-2022	7-2-2023	Wheat flour-sugar-hydrogenated vegetable oils-dextrose-fructose syrup-cocoa powder-milk powder- table salt-raising materials-preservatives-xanthan gum-moisturizing substances- food emulsifiers-vanillin.
13	8-8-2022	7-2-2023	Wheat flour-sugar-hydrogenated vegetable oils-dextrose-fructose syrup-cocoa powder-milk powder- table salt-raising materials-preservatives-xanthan gum-moisturizing substances- food emulsifiers-vanillin.
14	19-10-2022	18-1-2023	Wheat flour-sugar-fresh eggs- vegetable oils (refined palm oil–soybean oil–sunflower oil )–sorbitol (moisturizer and sweetener)–glucose syrup–fructose syrup–moisturizer (alimentary glycerin)–monopropylene glycerol) – natural strawberry-dried milk (bovine)-raising agents (E 500-E 50)-alimentary emulsifiers of origin plant-(E 471-E 475–E 481)-salt preservatives (potassium sorbate)-Thickener (xanthan gum guar gum-pectin E 262)-Vanillin-Citric acid -Healthy food color (red beetroot-red karmen)-aroma and flavor complaint with natural strawberry

^**⁎**^storage condition in supermarkets at room temperature

### Isolation and determination of fungal total number in cake samples

The dilution plate method recommended by Pitt and Hocking [8] was employed. For each sample, under aseptic conditions, ten grams of each cake were weighed and homogenized in 90 mL of 0.1% sterile peptone water (0.1 g of peptone per 100 mL of distilled water), followed by shaking for 15 minutes. Aliquots of 1 mL of sample suspension were aseptically transferred to sterile plates containing about 20 mL of solidifying agar medium, potato dextrose agar PDA, and malt extract agar MEA, and 3 plates for each sample. Petri dishes were rotated by hand in a circular motion so that the dilution was distributed in the agar medium. Petri dishes were incubated at 28^°^ C and 4° C for 7 – 10 days, after which the growing fungal colonies were identified and counted. The number of colonies per g in the original sample was estimated by the average number of colonies for each petri dish multiplied by the dilution factor.

### Identification of filamentous *fungi*

The morphological characteristics based on macroscopic features ( colony form and pigments) and microscopic appearance of hyphae and spores were used for identification of fungi up to genus and species levels. The following references were consulted for identification; Domsch et al. [24], for fungi in general, Moubasher [25], for soil fungi in Qatar and other Arab countries: Pitt [26], for *Penicillium* species: Pitt and Hocking [8], for food borne fungi; and Niaz and Dawar [27], for the genus *Aspergillus*. Fungi were identified at the mycology center, Faculty of Science, Assiut University.

### Production of extracellular enzymes

#### Amylase production

Twenty-seven isolates, including ;7 isolates of *A. flavus* group, 8 isolates of *A. niger* group, 1 isolate of *A. nidulans*, 1 isolate of *A. ochraceous*, 2 isolates of *A. terreus*, 1 isolate of *A. versicolor*, 1 isolate of *Cochlobilus lunatus*, 2 isolates of *Rhizopus stolonifer*, 1 isolate of *Trichoderma harzianum, and* 3 isolates of *Penicillium glabrum,* were tested using the starch hydrolysis medium Sivaramakrishnan et al. [28], which has the composition of (g/L) starch 15 g , K _2_H PO_4_ 1 g, MgSO_4_ .7 H_2_O 0.5 g and agar 15 g and distilled water to one liter. The medium was sterilized by autoclaving at 121^o^ C for 20 minutes, then 20 mL of medium was poured into sterilized petri dishes, after that plates were inoculated with equal discs of fungal isolates and incubated at 25°C for 3-4 days. Iodine solution was used as an indicator for starch analysis. The clear zone around the colony was measured.

### Protease production

Twenty-seven isolates, including: 7 isolates of *A. flavus* group, 8 isolates of *A. niger* group, 1 isolate of *A. nidulans*, 1 isolate of *A. ochraceous*, 2 isolates of *A. terreus*, 1 isolate of *A. versicolor*, 1 isolate of *Cochlobilus lunatus*, 2 isolates of *Rhizopus stolonifer*, 1 isolate of *Trichoderma harzianum,* and 3 isolates of *Penicillium glabrum,* were tested using the casein hydrolysis medium Paterson and Bridge [29], which contains of (g/l): KH_2_ PO_4_, 1.0; KCl, 0.5; MgSO_4_.7 H_2_O, 0.2; CaCl_2_.2H_2_O, 0.1; 15% skimmed milk, 25 mL; glucose , 10 agar, 15; distilled water to one liter. The medium was distributed into 20 mL test tubes (10 mL/tube), sterilized by autoclaving at 121^o^ C for 20 minutes. Tubes containing media were individually inoculated with the tested fungal isolates and incubated at 25^o^ C for 7 days. The complete degradation of milk protein was seen as clear depth in the tube. The clear depth below the colony was measured in mm.

### Lipase production

Twenty-seven isolates, including 7 isolates of the *A. flavus* group, 8 isolates of *A. niger* group, 1 isolate of *A. nidulans*, 1 isolate of *A. ochraceous*, 2 isolates of *A. terreus*, 1 isolate of *A. versicolor*, 1 isolate of *Cochlobilus lunatus*, 2 isolates of *Rhizopus stolonifer*, 1 isolate of *Trichoderma harzianum,* and 3 isolates of *Penicillium glabrum,* were tested using Phenol red [30]: which was performed in agar plates containing 0.01 g of phenol red, 0.1 g of CaCl_2_ 5 H_2_ O , 2 g of olive oil, and 2 g of agar in 100 mL distilled water. The different strains are incubated, and the halo zone diameter is measured in mm.

### Detection of mycotoxins from cake samples

Analysis of mycotoxins was performed using liquid chromatography–electrospray ionization tandem mass spectrometry (LC-ESI-MS/MS) with an ExionLC AC system for separation and a SCIEX Triple Quad 5500 + MS/MS system equipped with an electrospray ionization (ESI) for detection. The instrument data were collected and processed using the SCIEX OS 1.6.10.40973 software [31] (Table 2).

**Table 2:** Multiple Reaction Monitoring (MRM) parameters for the determination of aflatoxins and ochratoxin A

**Q1 (m/z)**	**Q3 (m/z)**	**RT (min)**	**ID**	**DP (V)**	**EP (V)**	**CE (V)**	**CXP (V)**
404.2	239	11.03	OTA 404.2/239	50	10	30	3
404.2	221	11.03	OTA 404.2/221	50	10	39	3
313.04	285	8.78	AFB1 313.040/285	116	10	33	16
313.04	241.1	8.78	AFB1 313.040/241.1	116	10	51	14
315.2	287.1	8.49	AFB2 315.2/287.1	91	10	39	14
315.2	259.1	8.49	AFB2 315.2/259.1	91	10	45	22
329.017	243	8.09	AFG1 329.017/243	136	10	37	14
329.017	311	8.09	AFG1 329.2/311	136	10	31	18
331.2	313	7.76	AFG2 331.2/313	131	10	36	14
331.2	245	7.76	AFG2 331.2/245	131	10	49	18

(Rt) retention times, (Q1) the m/z of precursor ion, (Q3) the m/z of monitored product ions, CE (V) collision energy, CXP (V) collision cell exit potential, DP (V) linear sweep differential pulse, EP (V) Entrance potential

The separation of the targeted analytes was performed with an Agilent Zorbax Eclipse Plus C18 Column (4.6×100 mm, 1.8 µm). The mobile phases consisted of two eluents, both containing 10 mM ammonium formate; A was 0.1% formic acid in water, and eluent B was 0.1% formic acid in methanol (LC grade). The mobile phase gradient was programmed as follows: 10% B at 0 min, 10-30% B from 0.0-2.0 min, 30-100% B from 2.0-11.0 min, 100% B from 11.0-11.5 min, 100-10% B from 11.5-12.0 min, 10% B from 12.0 to 15.0 min. The flow rate was 0.6 mL/min, and the injection volume was 10 µL. For MS/MS analysis, positive ionization mode (+MRM) was applied with the following parameters: curtain gas: 20 psi; collision gas: 9 psi; nebulizer current: 3; source temperature: 600°C; ion source gas 1 (nebulizer gas): 60 psi.

### Cake Samples extraction

Two grams of sample were extracted by 8 mL of extraction solution (ACN:Water, 80:20), vortexed for 20 min, centrifuged, and then filtered using a 0.45 uM filter. Finally, 0.5 mL of the filtrate was diluted 1:1 with 5 mM ammonium acetate.

### Effect of natural oils (Clove, Peppermint and Olive ) on the growth of the isolated *fungi* by well diffusion method

Clove, peppermint, and olive oil were tested in this study because of their higher abilities as antifungal agents. Twenty-seven isolates 7 isolates of the *A. flavus* group, 8 isolates of the *A. niger* group, 1 isolate of *A. nidulans*, 1 isolate of *A. ochraceous*, 2 isolates of *A. terreus*, 1 isolate of *A. versicolor*, 1 isolate of *Cochlobilus lunatus*, 2 isolates of *Rhizopus stolonifer*, 1 isolate of *Trichoderma harzianum,* and 3 isolates of *Penicillium glabrum* were tested in this study. The cup plate technique recommended by Staib et al. [32] was employed with some modifications using PDA medium for growing the fungal isolates. The medium contained (g/L) potato, 200 g; glucose, 20 g; agar, 20 g; and distilled water, 1000 mL. Spore suspension (1 mL) of each test fungus, was transferred into a petri dish and poured under aseptic conditions. Cooled PDA medium was poured in the petri dishes over the spore suspension and then mixed together by moving the petri dishes clock-wise and anti-clock-wise. Using a sterile cork porer , one cavity was made in the petri dish and filled with 100 µL of clove oil, 100 µL of the peppermint oil and 100 µL of olive oil . All petri dishes were incubated at 28^o^ C for 4 days. The diameters of the inhibition zone (mm) were measured at the end of incubation period.

### Statisitical analysis

Statistical analysis was done using SPSS (statistical software programs, predicative analysis, predicative analytics and decision support systems) (version 10) (Real State, Real Easy).

## Results

### Isolation of *fungi* from cake samples

A total of 5 species belonging to 3 genera were recovered from cake samples on potato dextrose agar medium (PDA), and 8 species belonging to 4 genera were recovered from malt extract agar medium (MEA), as shown in Tables 3 and 4.

**Table 3:** Total fungal count (colonies/ g) associated with 14 cake samples recovered on PDA medium

**Genera and species**	**Sample number**														**Total count**	**% of total count**	**No. of cases of isolation**
	**1**	**2**	**3**	**4**	**5**	**6**	**7**	**8**	**9**	**10**	**11**	**12**	**13**	**14**			
***Aspergillus***	3.33	0	0	0	0	3.33	3.33	6. 66	0	0	0	3.33	0	16. 66	36. 64	84. 61	6
***A .flavus***	0	0	0	0	0	0	0	6. 66 ± 0.57	0	0	0	0	0	6. 66 ± 0.57	13.32	30.76	2
***A. niger***	3.33 ± 0.57	0	0	0	0	3.33 ± 0.57	3.33 ± 0.57	0	0	0	0	0	0	10 ±0.00	19.99	46.16	4
***A .nidulans***	0	0	0	0	0	0	0	0	0	0	0	3.33 ± 0.57	0	0	3.33	7. 69	1
***Cochlobilus lunatus***	0	0	0	0	0	0	0	0	0	3.33 ± 0.57	0	0	0	0	3.33	7. 69	1
***Penicillium glabrum***	0	0	0	0	0	0	0	0	0	0	3.33	0	0	0	3.33	7. 69	1
**Total count**															43.3		
**No. of genera**															3		
**No.of species**															5

**Table 4:** Total fungal count (colonies / g) associated with 14 cake samples recovered on MEA medium

**Genera and species**	**Sample number**														**Total count**	**% of total count**	**No. of cases of isolation**
	**1**	**2**	**3**	**4**	**5**	**6**	**7**	**8**	**9**	**10**	**11**	**12**	**13**	**14**			
***Aspergillus***	23.33	3.33	3.33	13.32	0	13.32	0	6. 66	6. 66	9.99	6. 66	10	0	6. 66	103.26	83.78	11
***A. flavus***	3.33 ±0.57	0	3.33 ±0.57	3.33 ±0.57	0	3.33 ±0.57	0	0	0	0	0	10 ±1.73	0	3.33 ±0.57	26.65	21.62	6
***A. niger***	20 ±2.6**4**	0	0	6. 66 ±0.57	0	6. 66 ±1.15	0	6. 66 ±1.15	6. 66 ±0.57	6. 66 ±0.57	6. 66 ±0.57	0	0	3.33 ±0.57	63.29	51.35	8
***A. ochraceous***	0	3.33 ±0.57	0	0	0	0	0	0	0	0	0	0	0	0	3.33	2.70	1
***A. terreus***	0	0	0	3.33 ±0.57	0	0	0	0	0	3.33 ±0.57	0	0	0	0	6. 66	5. 40	2
***A. versicolor***	0	0	0	0	0	3.33 ±0.57	0	0	0	0	0	0	0	0	3.33	2.70	1
***Penicillium glabrum***	0	0	3.33 ±0.57	0	0	6. 66 ±1.15	0	0	0	0	0	0	0	0	9.99	8.10	2
***Rhizopus stolonifera***	0	0	0	0	0	0	0	0	0	0	3.33 ±0.57	3.33 ±0.57	0	0	6. 66	5. 40	2
***Trichoderma harzianum***	0	0	0	3.33 ±0.57	0	0	0	0	0	0	0	0	0	0	3.33	2.70	1
**Total count**															123.24		
**No. of genera**															4		
**No. of species**															8		

On PDA, a total of 5 species assigned to 3 genera were isolated from cake samples on PDA medium, as shown in Table 3. Fungi were identified in the mycology center, Faculty of Science, Assiut University. The fungal total count was (43.3 colonies/g). *Aspergillus* was the most dominant genus and was isolated from 6 samples of cake. *Aspergillus* was represented by 3 species, namely, *A. flavus*, *A. niger,* and *A. nidulans* represented 13.32, 19.99, and 3.33 Colonies/g, respectively. The most frequent species was *A. niger,* which isolated from 4 samples, and the percentage of the total count was 46.16 %. *Cochlobilus luntaus* and *Penicillium glabrum* were isolated from one sample only, represented by (3.33 and 3.33 Colonies/g) respectively.

On Malt Extract Agar Medium (MEA), a total of 8 species assigned to 4 genera were isolated from cake samples on MEA medium, as shown in Table 4. The fungal total count was (123.24 colonies/g*). Aspergillus* was the most dominant genus and was isolated from 11 samples of cake. *Aspergillus* was represented by 5 species, namely*, A. flavus*, *A. niger*, *A. ochraceous*, *A. terreus,* and *A. versicolor,* represented by 26.65, 63.29, 3.33, 6.66 and 3.33 colonies/g, respectively. The most frequent species was *A. niger,* which was isolated from 8 samples, and its total count percentage was 51.35 %. *Penicillium glabrum* and *Rhizopus stolonifer* were isolated from 2 samples represented by 8.10 and 5.40 colonies/g, respectively. *Trichoderma harzianum* was isolated from 1 sample, represented by 2.70 colonies/g.

### Enzymes production

#### Amylase production

**Fig. 1:**
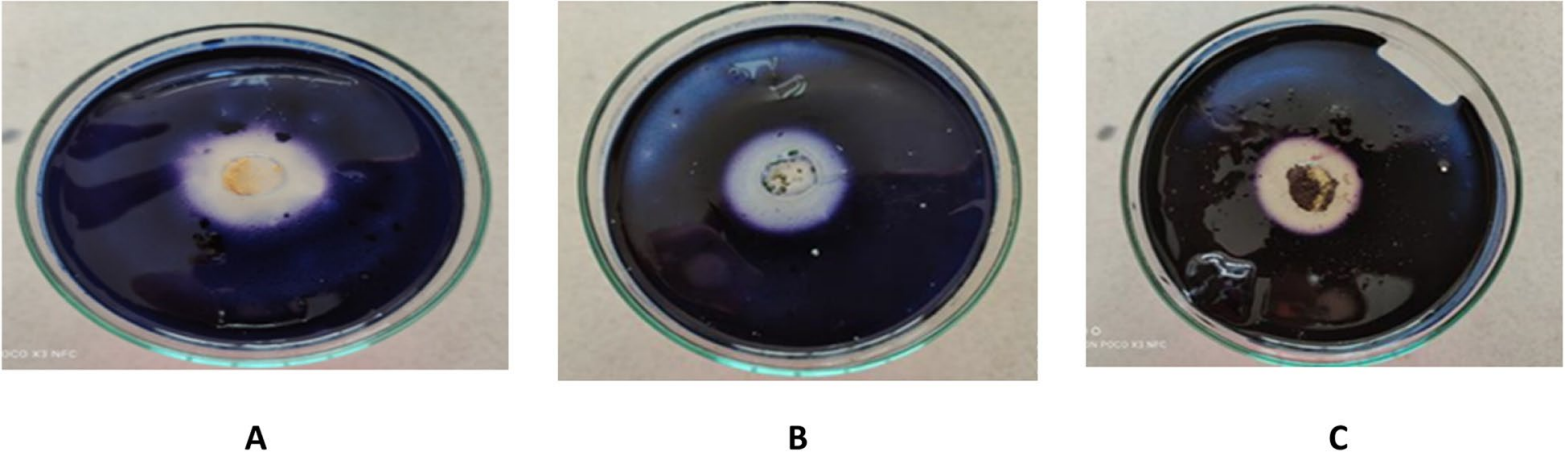
Amylase production by some filamentous fungal isolates. (notice the clear zone). **A** *Aspergillus ochraceous* that isolated from sample no. 2. **B** *Trichoderma harzianum* that isolated from sample no. 4. **C** *Aspergillus niger* that isolated from sample no. 1

Results from Table 5 and Fig. 1 show the ability of 27 fungal isolates to production of amylase enzyme. Twenty-four isolates (88.88%) of the total twenty-seven tested filamentous fungi showed positive results. The highest enzyme production was detected in nine samples, and the clear zone ranged from 29.3-60.8 mm. Moderate enzyme production was detected in 13 samples, and the clear zone ranged from 18.8-28.9 mm. The low enzyme production was detected in 2 samples *A. niger,* which was isolated from sample no. 11, and *A. terreus,* that isolated from sample no.4 and showed a clear zone of 11.1 and 10.1 mm, respectively.

**Table 5:** Production of Amylase, lipase and protease qualitatively by the isolated fungi from cake samples

**Fungal isolates**	**Source**	**Amylase activity (mm)**		**lipase activity (mm)**		**Protease activity (mm)**	
			**Levels**		**Levels**		**levels**
***Aspergillus***							
***A.flavus***	Sample no. 1	51.9±0.20	H	-	-	-	-
***A.flavus***	Sample no. 3	44.4±0.08	H	-	-	-	-
***A.flavus***	Sample no. 4	54.5±0.24	H	-	-	-	-
***A.flavus***	Sample no. 6	47±0.30	H	-	-	22.5±0.07	M
***A.flavus***	Sample no. 8	53±0.40	H	-	-	-	-
***A.flavus***	Sample no. 12	60.8±0.34	H	-	-	-	-
***A.flavus***	Sample no. 14	55.8±0.36	H	-	-	-	-
***A. nidulans***	Sample no. 12	20±0.10	M	-	-	-	-
***A.niger***	Sample no. 1	26.4	H	18.9±0.06	M	-	-
***A.niger***	Sample no. 4	23.9±0.20	M	26.1±0.26	M	-	-
***A.niger***	Sample no. 6	28.9±0. 61	M	25.1±0.07	M	-	-
***A.niger***	Sample no. 7	24±0.10	M	10±0.00	L	-	-
***A.niger***	Sample no. 8	18.8±0.10	M	18.1±0.00	M	-	-
***A.niger***	Sample no. 9	20.4±0.22	M	18.3±0.08	M	23±0.00	M
***A.niger***	Sample no. 11	11.10.10	L	20.9±0.18	M	10.5±0.07	L
***A.niger***	Sample no. 14	23.1±1.8	M	10±0.00	L	-	-
***A. ochraceous***	Sample no. 2	27.9±0.27	M	-	-	10.5±0.07	L
***A.terreus***	Sample no. 4	10.1±0.04	L	-	-	19±0.14	M
***A. terreus***	Sample no. 10	20.1±0.09	M	-	-	-	-
***A. versicolor***	Sample no. 6	28.9±0.07	M	-	-	24±0.00	M
***Cochlobilus lunatus***	Sample no. 10	22.3±0.19	M	22.9±0.18	M	-	-
***Rhizopus stolonifera***	Sample no. 11	29.3±0.12	H	15.1±0.07	M	15.5±0.07	M
***Rhizopus stolonifer***	Sample no. 12	21.6±0.33	M	-	-	-	-
***Trichoderma harzianum***	Sample no. 4	27.6±0.16	M	-	-	14.5±0.07	L
***Penicillium***				-	-		
***P. glabrum***	Sample no. 3	-	-	-	-	24. 6±0.35	M
***P. glabrum***	Sample no. 6	-	-	-	-	30±0.10	H
***P. glabrum***	Sample no. 11	-	-	-	-	24.6±0.05	M

Degree of activity *H* high (30-60), *M* moderate (15-29), *L* low (≤ 15)

#### Lipase production

**Fig. 2:**
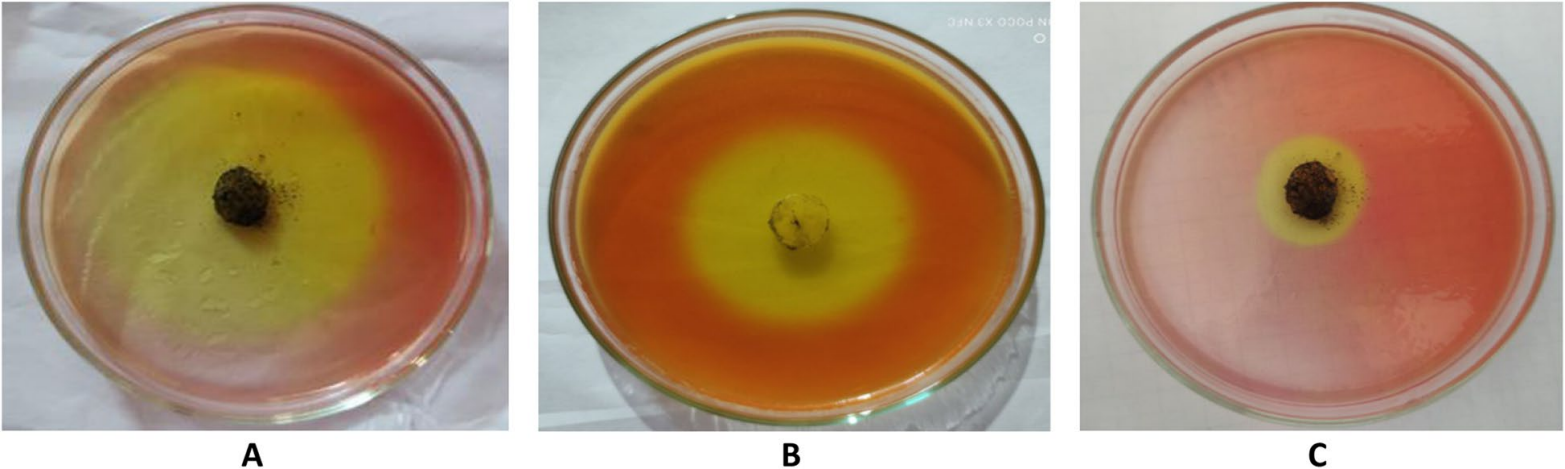
Lipases production by some fungal isolates (notice the clear zone). **A** *Aspergillus niger* that isolated from sample no. 9. **B** *Rhizopus stolonifer* that isolated from sample no. 11. **C** *Aspergillus niger* that isolated from sample no. 11

Results in Table 5 and Fig. 2 show the ability of 27 fungal isolates in produce of lipases enzymes. Ten isolates (37.03 %) of the tested 27 filamentous fungi showed positive results. Moderate enzyme production was detected in 8 samples, and the clear zone ranged from 15.1 to 26.1 mm. Low enzyme production was showen by 2 isolates of *A. niger* that were isolated from sample no. 7 and *A. niger* that was isolated from sample no. 14, whereas, the clear zone was 10 mm for both. On the other hand , 17 isolates (63 % of the total tested filamentous fungi) could not produce lipase enzymes (Table 5).

#### Protease production

**Fig. 3:**
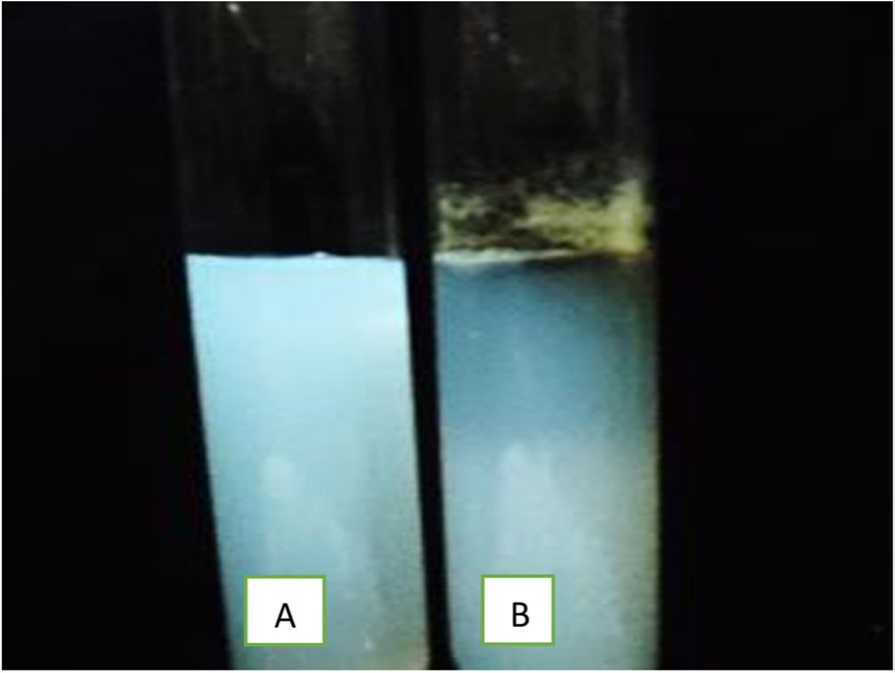
Protease production by *Aspergillus versicolor*. **A** Control, **B**: *Aspergillus versicolor*

Also, 27 isolates of the total tested filamentous fungi were examined for their ability to produce proteases enzyme production Table 5 and Fig. 3). Eleven isolates (40.74%) showed positive results. The highest enzyme production was detected by *Penicillium glabrum,* that isolated from sample no. 6 represented by 30 mm. Seven isolates (25.92 %) of them were moderate in protease production and the depth of the clear zone ranged from 15.5 to 24.6 mm. Two isolates were low producers of proteases ,identified as *A. ochraceous* from sample no. 2 and *Trichoderma harzianum* from sample no. 4, and represented clear zones of 10.5 and 14.5 mm, respectively.

### *Detection of* mycotoxins from cake samples

Aflatoxins B_1_, B_2_, G_1_, G_2_ and Ochratoxin A were not detected in fourteen samples of cake (Figs. 4, 5, 6, 7, 8, 9, 10, 11, 12, 13, 14, 15, 16, 17).

**Fig. 4:**
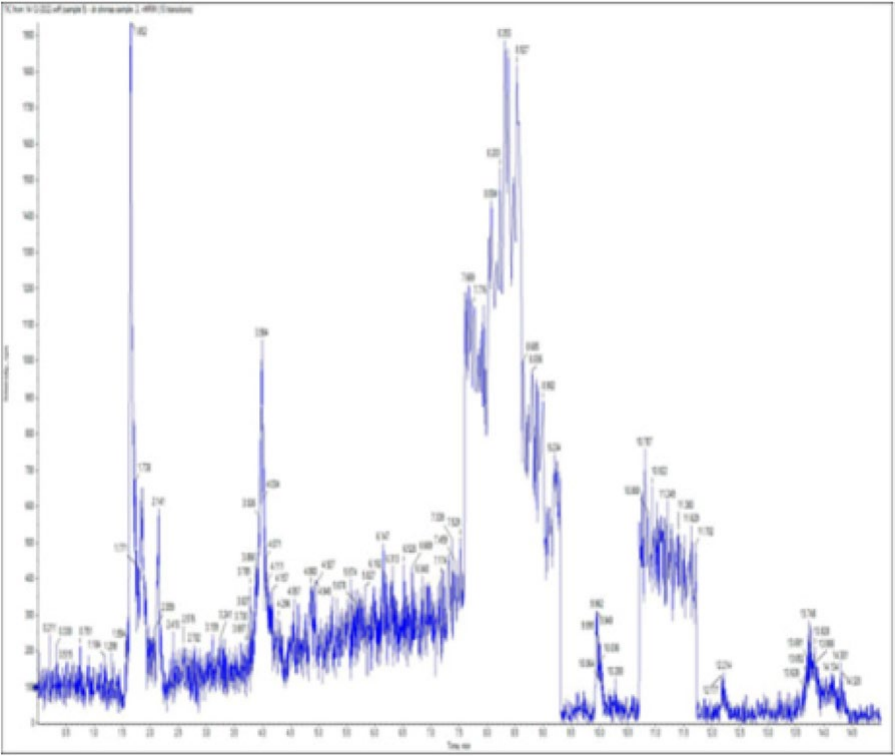
No mycotoxins indicated from cake sample no. 1

**Fig. 5:**
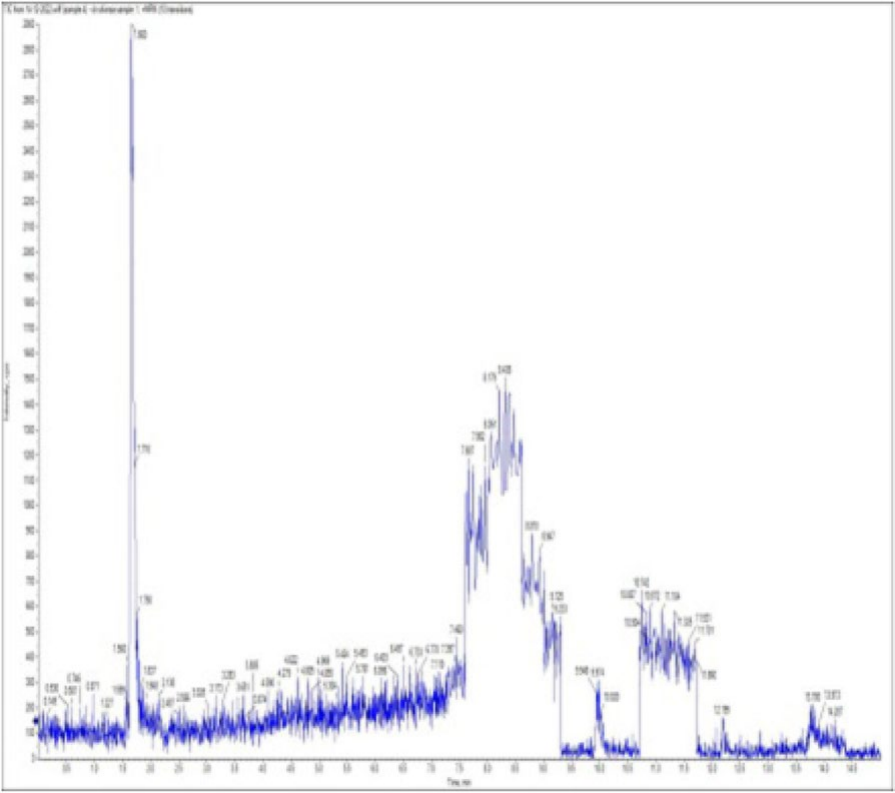
No mycotoxins indicated from cake sample no. 2

**Fig. 6:**
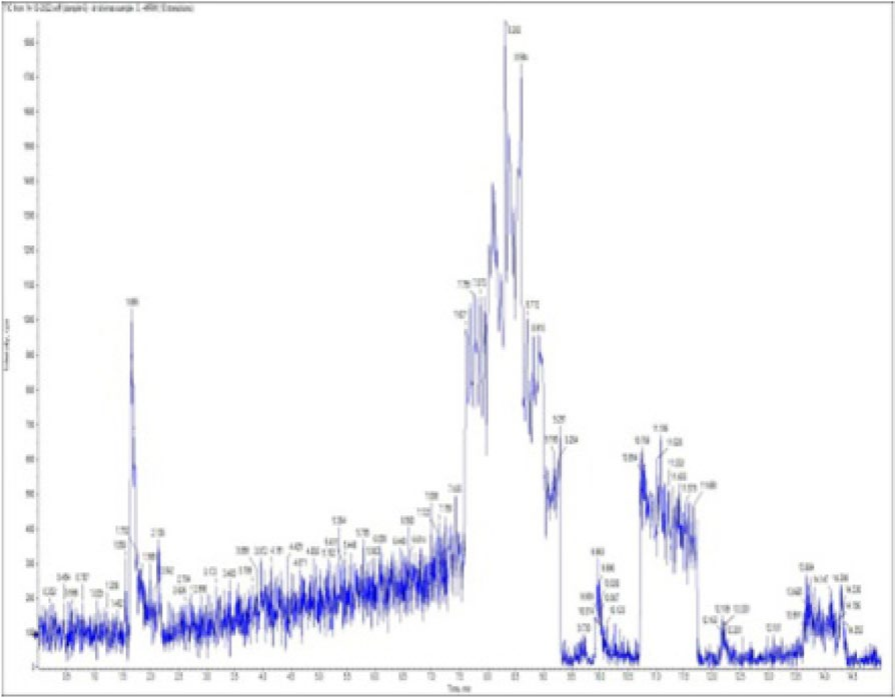
No mycotoxins detected from cake sample no. 3

**Fig. 7:**
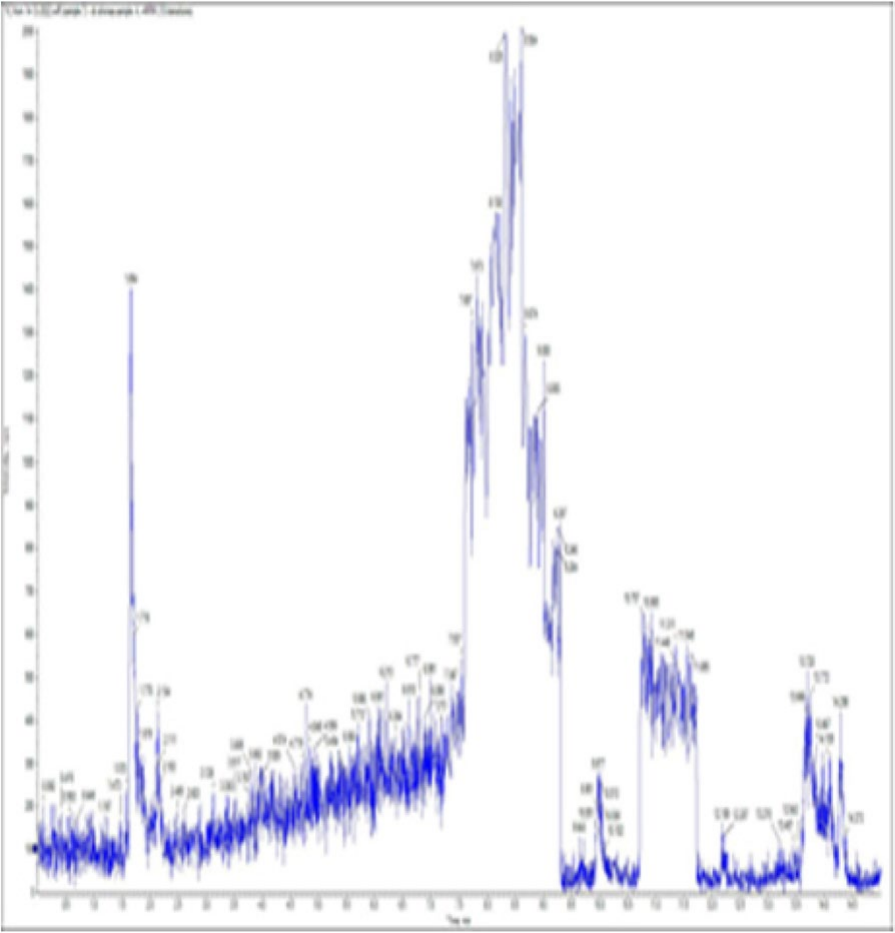
No mycotoxins detected from cake sample no. 4

**Fig. 8:**
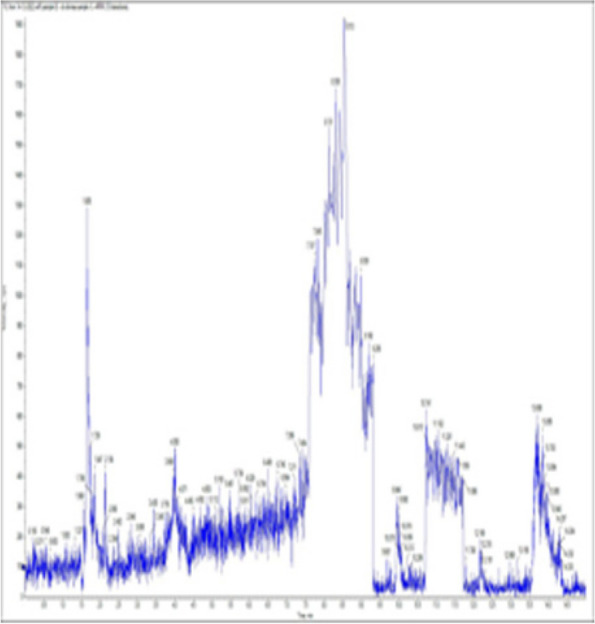
No mycotoxins detected from sample no. (5)

**Fig. 9:**
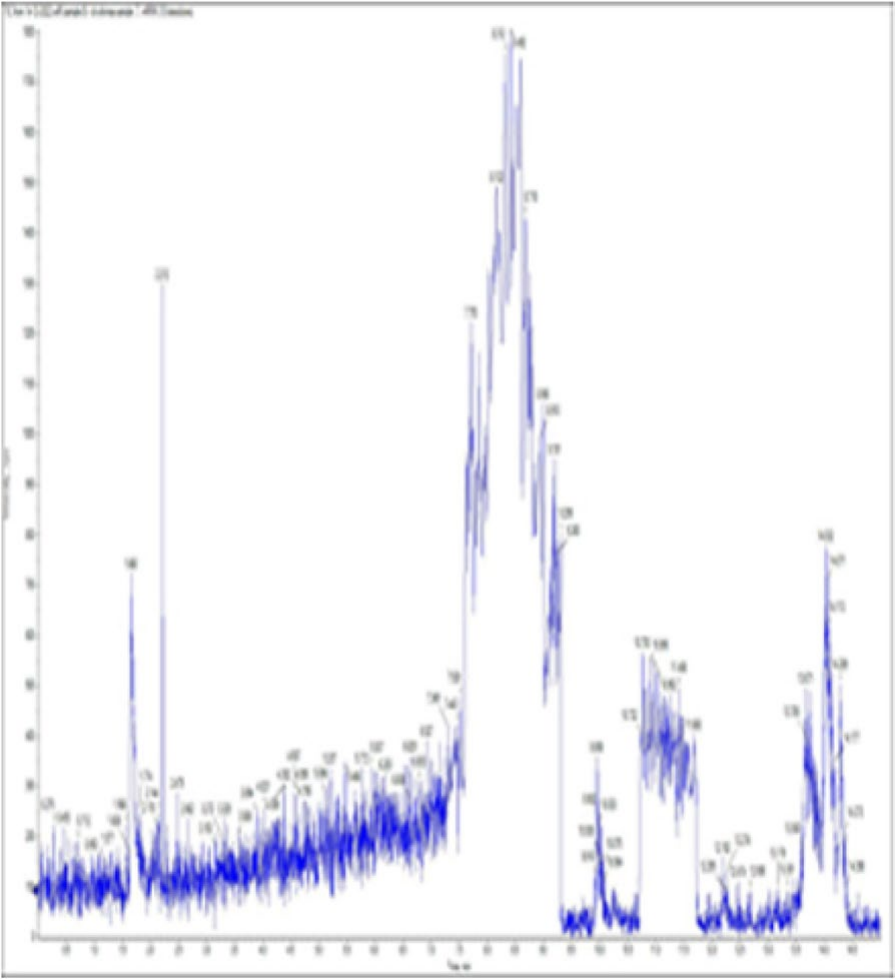
No mycotoxins detected from sample no. 6

**Fig. 10:**
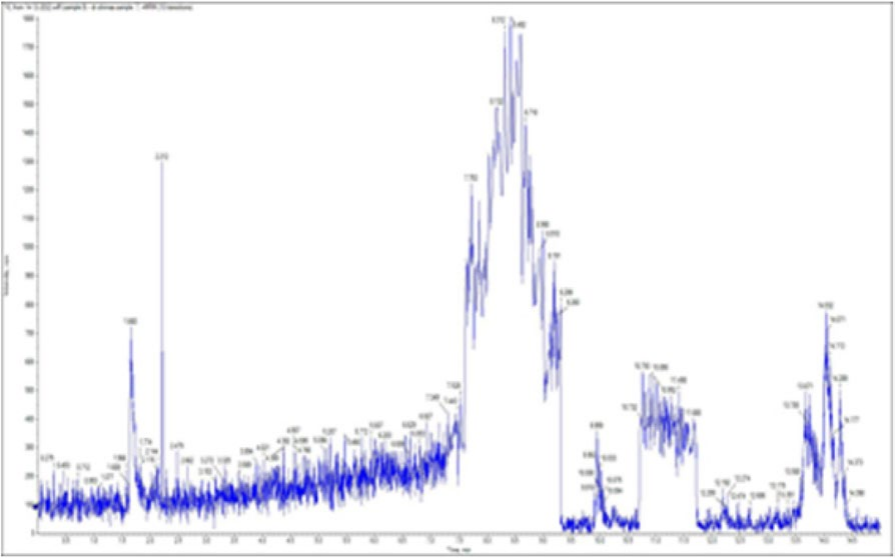
No mycotoxins detected from sample no. (7)

**Fig. 11:**
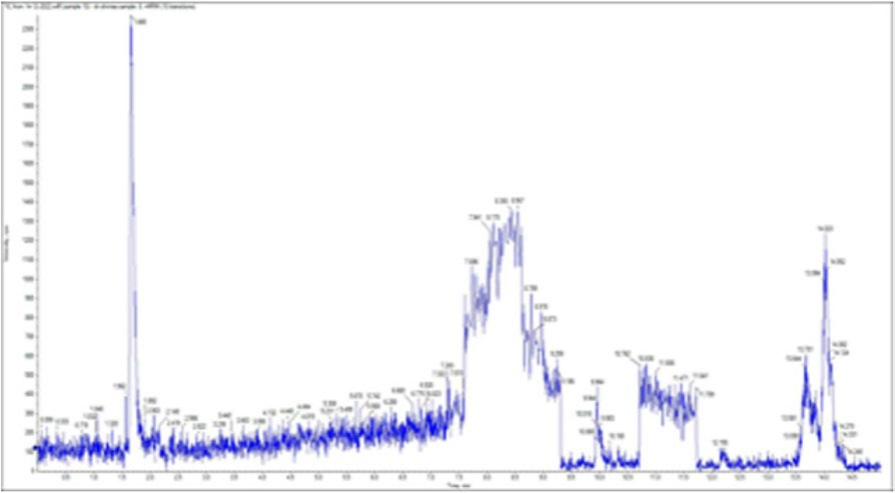
No mycotoxins detected from sample no. (8)

**Fig. 12:**
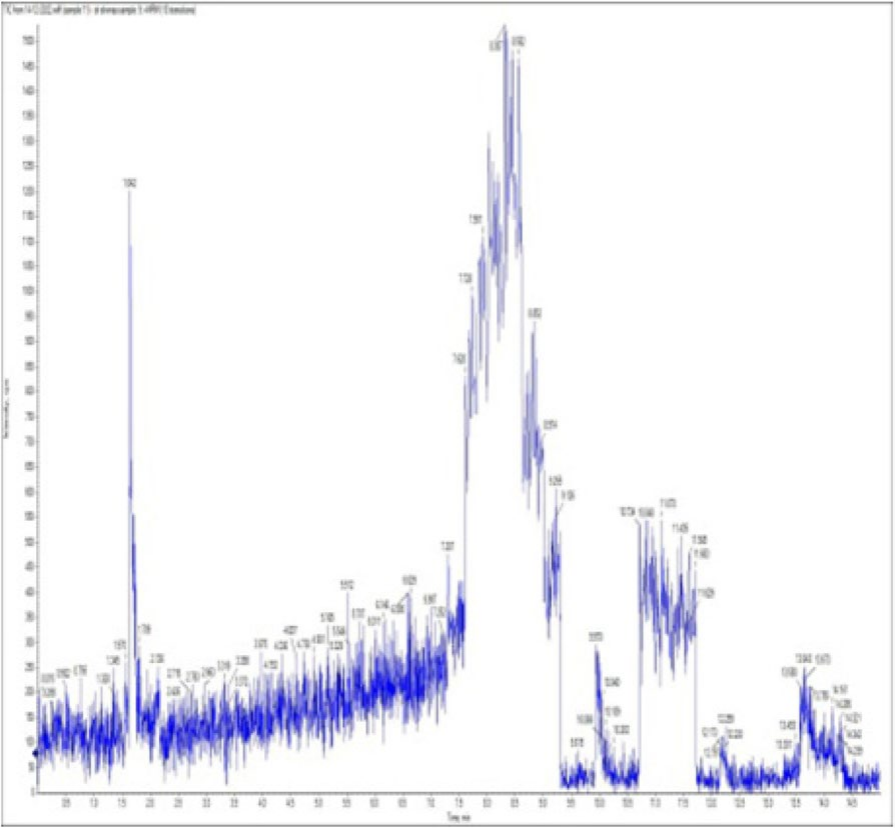
No mycotoxins detected from sample no. (9)

**Fig. 13:**
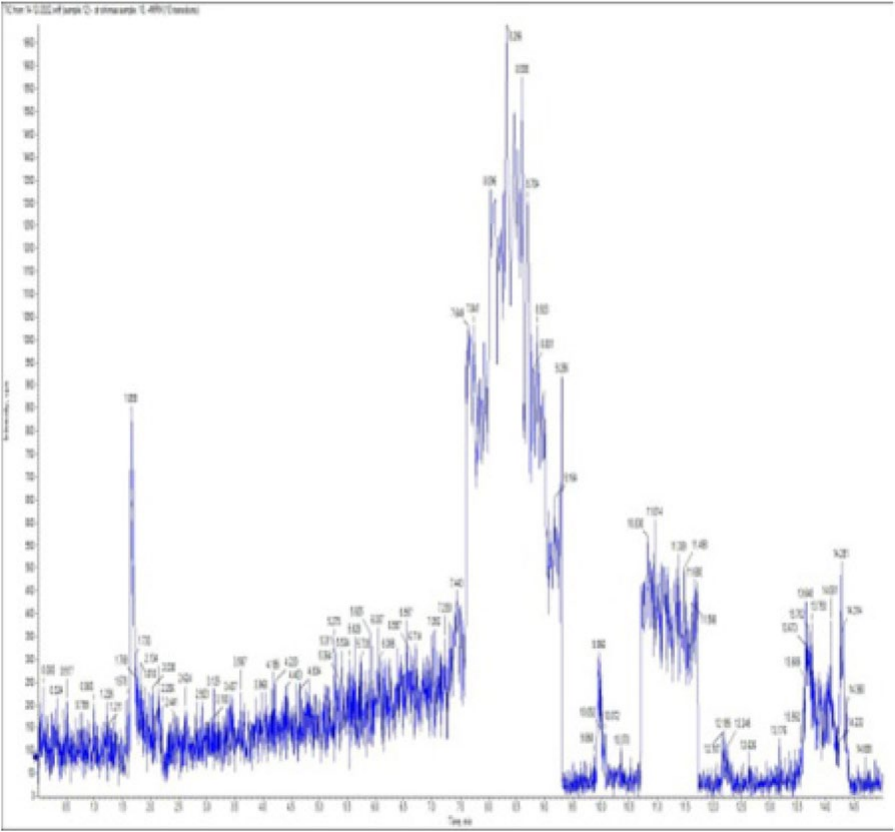
No mycotoxins detected from sample no. (10)

**Fig. 14:**
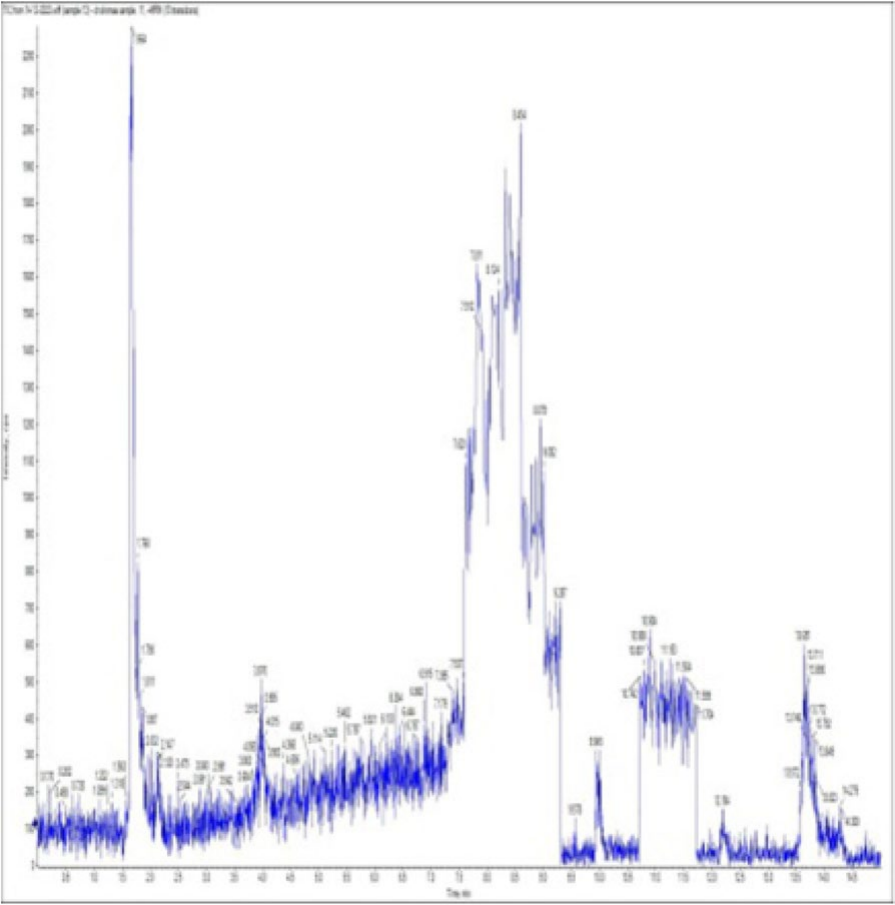
No mycotoxins detected from sample no. (11)

**Fig. 15:**
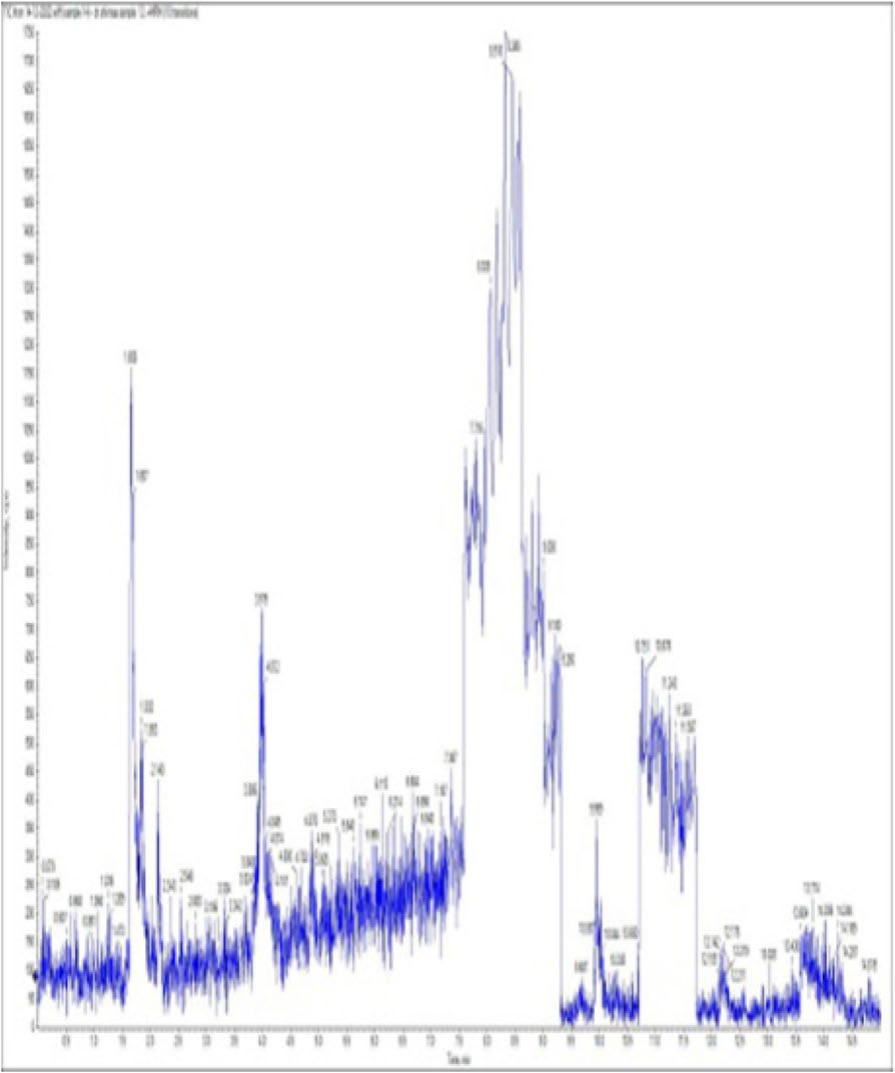
No mycotoxins detected from sample no. (12)

**Fig. 16:**
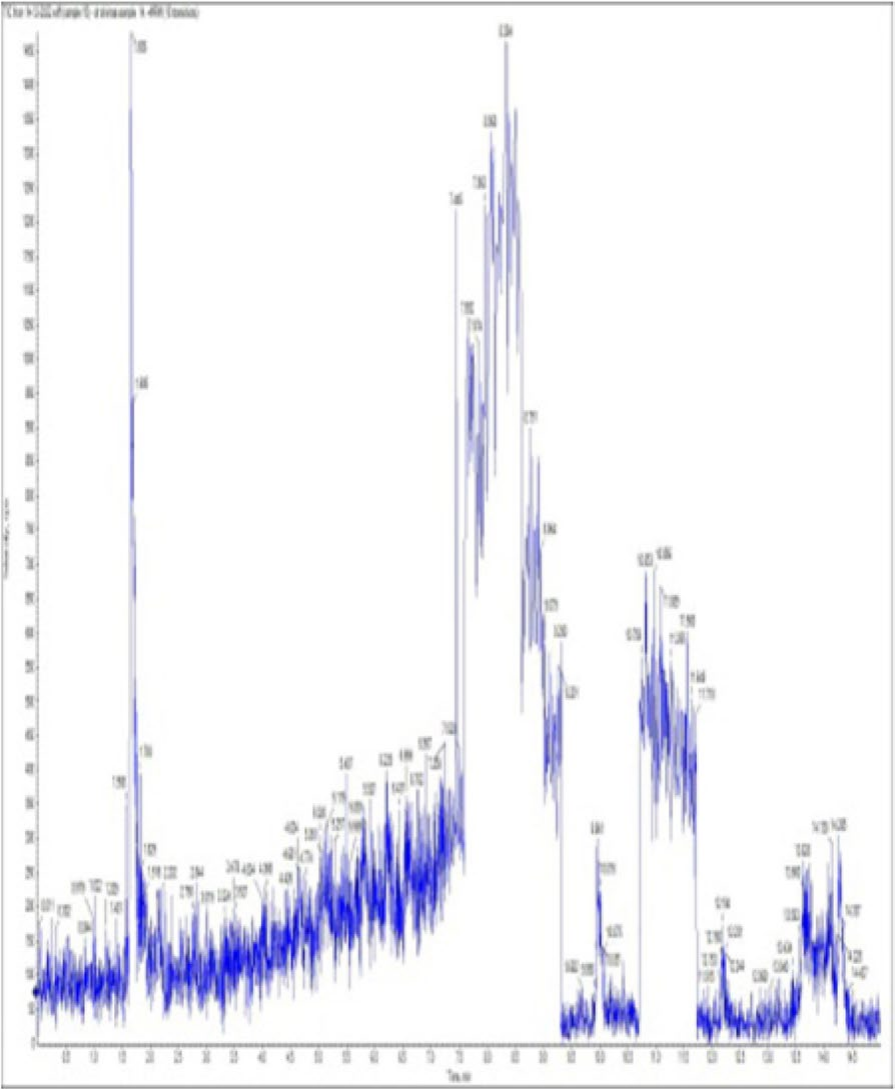
No mycotoxins detected from sample no. (13)

**Fig. 17:**
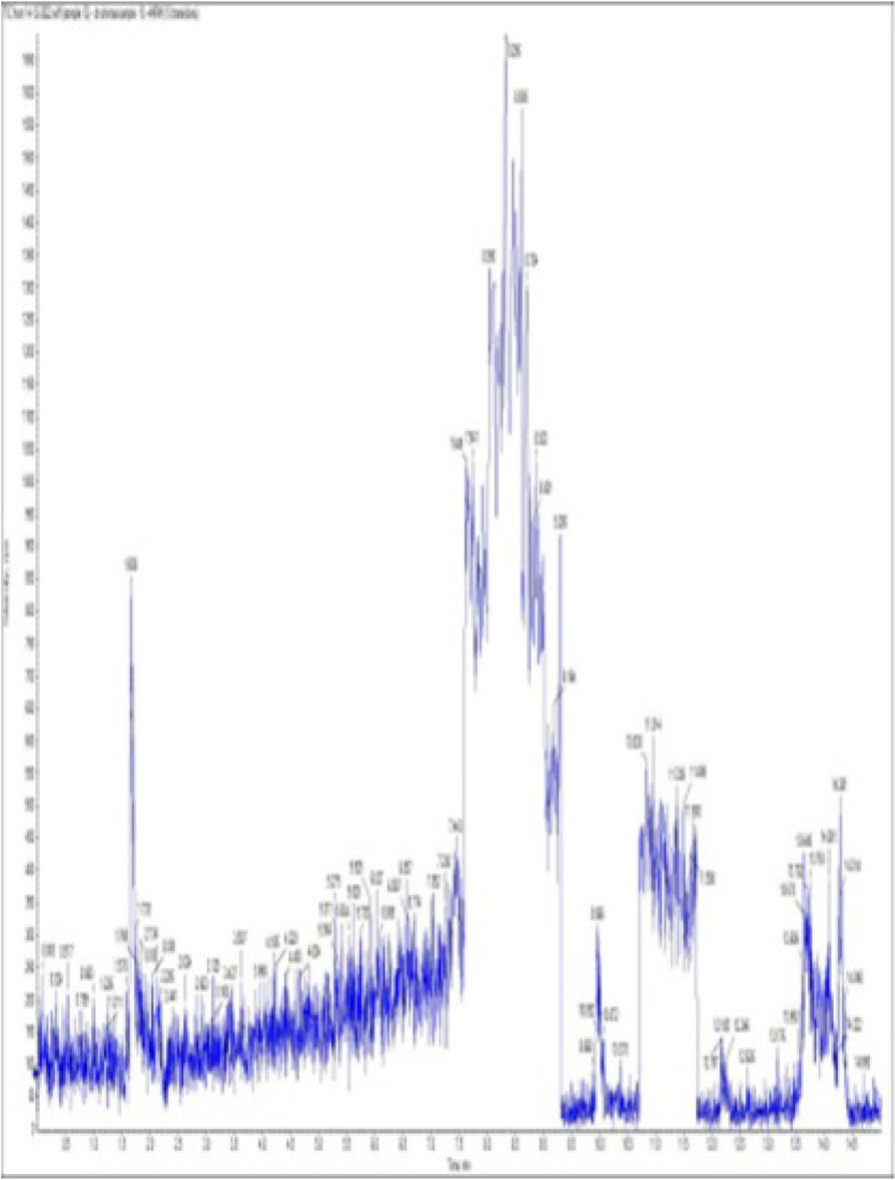
No mycotoxins detected from sample no. (14)

### Effect of natural oils (Clove, Peppermint and Olive) on the growth of the isolated *fungi* by well diffusion

**Fig. 18:**
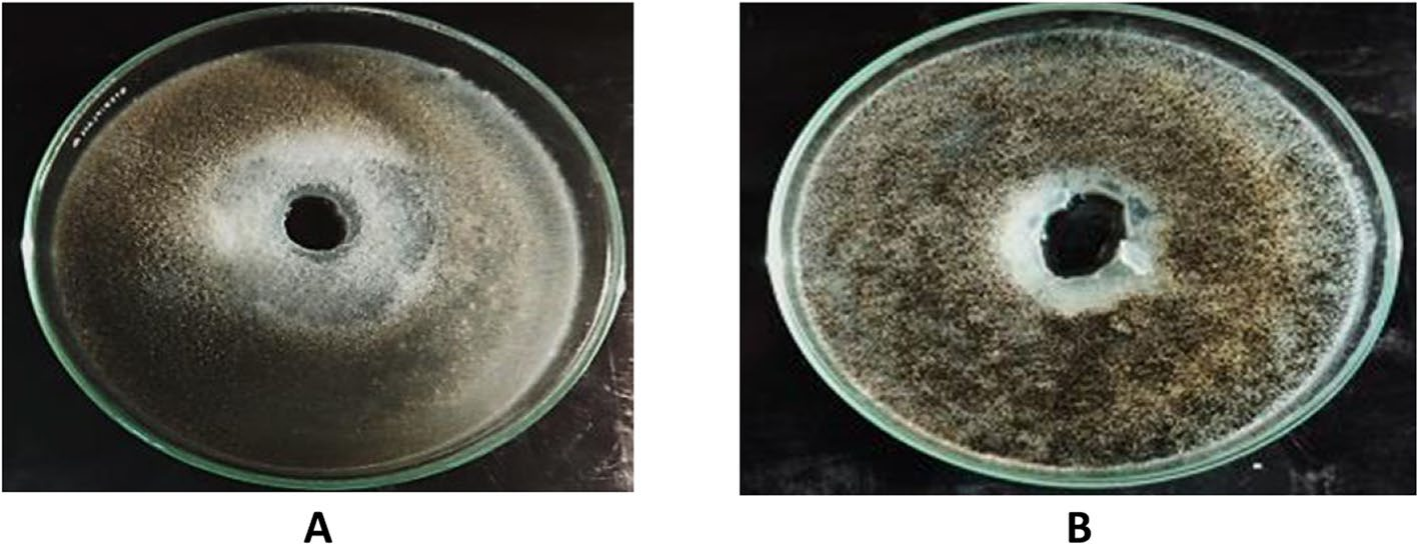
Antifungal activity of clove oil against A-*Rhizopus stolonifer* that isolated from sample no. 11, B-*A. niger* that isolated from sample no. 11

**Fig. 19:**
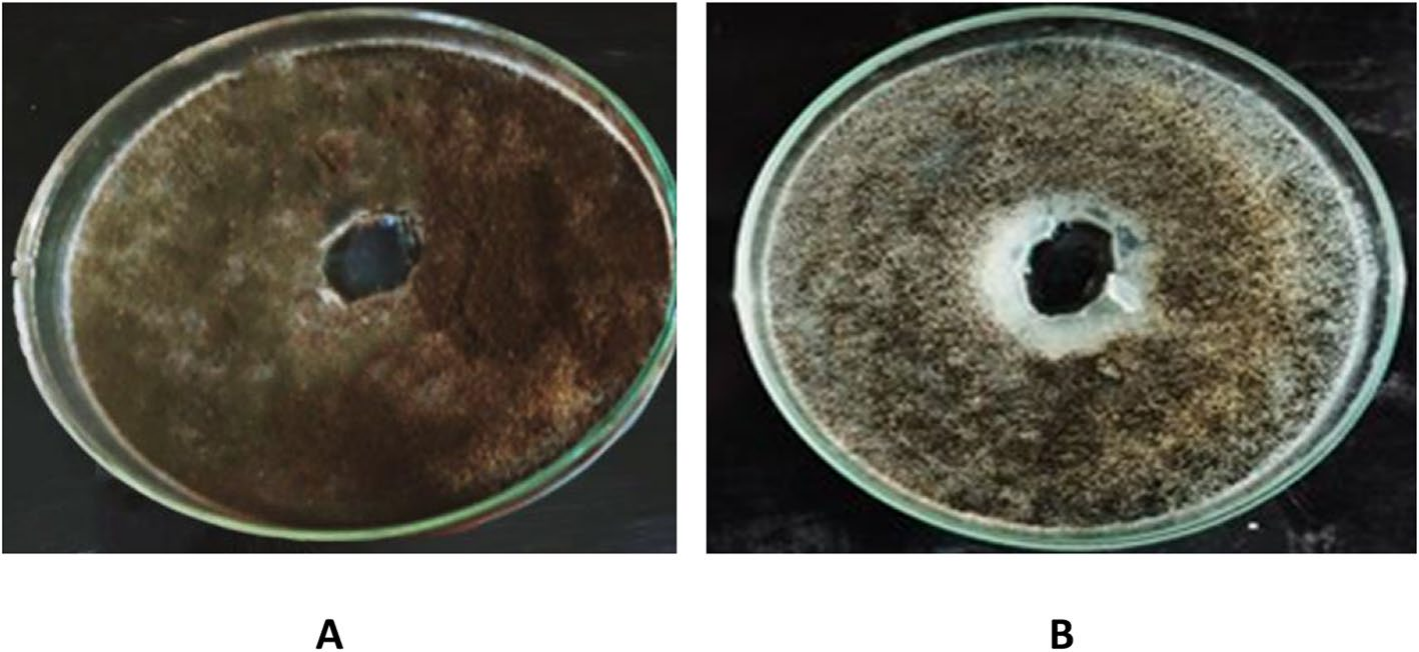
Antifungal Activity of A-Peppermint oil (no inhibition) and B-clove oil against *niger* that isolated from sample no. 11 (inhibition appeared)

A total of 27 isolates of filamentous fungi isolated from cake samples were examined for their sensitivity to each of clove, peppermint, and olive oil (Table 6 and Figs. 18 and 19).

**Table 6:** Inhibitory effect of natural oils on the growth of isolated fungi from cake samples

**Fungal isolates**	**Source**	**Clove oil (100 µL / well)**		**Peppermint oil (100 µL /well)**		**Olive oil (100 µL /well)**	
		**Inhibition zone (mm)**	**Sensitivity levels**	**Inhibition zone (mm)**	**Sensitivity levels**	**Inhibition zone (mm)**	**Sensitivity Levels**
***Aspergillus***							
*** A.flavus***	Sample no. 1	-	NS	-	NS	-	NS
*** A.flavus***	Sample no. 3	-	NS	-	NS	-	NS
*** A.flavus***	Sample no. 4	-	NS	-	NS	-	NS
*** A.flavus***	Sample no. 6	-	NS	-	NS	-	NS
*** A.flavus***	Sample no. 8	4±0.07	M	-	NS	-	NS
*** A.flavus***	Sample no. 12	3±0.00	M	-	NS	-	NS
*** A.flavus***	Sample no. 14	-	NS	-	NS	-	NS
*** A.nidulans***	Sample no. 12	1±0.00	M	-	NS	-	NS
*** A.niger***	Sample no. 1	1±0.00	M	-	NS	-	NS
*** A.niger***	Sample no. 4	3±0.07	M	-	NS	-	NS
*** A.niger***	Sample no. 6	-	NS	-	NS	-	NS
*** A.niger***	Sample no. 7	-	NS	-	NS	-	NS
*** A.niger***	Sample no. 8	5±0.00	M	-	NS	-	NS
*** A.niger***	Sample no. 9	-	NS	-	NS	-	NS
*** A.niger***	Sample no. 11	10±0.07	H	-	NS	-	NS
*** A.niger***	Sample no. 14	5±0.00	M	-	NS	-	NS
*** A. ochraceous***	Sample no. 2	5±0.00	M	-	NS	-	NS
*** A.terreus***	Sample no. 4	-	NS	-	NS	-	NS
*** A. terreus***	Sample no. 10	-	NS	-	NS	-	NS
*** A. versicolor***	Sample no. 6	-	NS	-	NS	-	NS
*** Cochlobilus lunatus***	Sample no. 10	-	NS	-	NS	-	NS
*** Rhizopus stolonifer***	Sample no. 11	19±0.07	H	-	NS	4±0.00	M
*** Rhizopus stolonifer***	Sample no. 12	-	NS	-	NS	-	NS
*** Trichoderma harzianum***	Sample no. 4	-	NS	-	NS	-	NS
***Penicillium***							
*** P. glabrum***	Sample no. 3	-	NS	3±0.00	M	-	NS
*** P. glabrum***	Sample no. 6	6±0.07	H	-	NS	-	NS
*** P. glabrum***	Sample no. 11	3±0.07	M	-	NS	2±0.00	M

*HS* High sensitive( ≥ 6 ), *MS* Moderate sensitive (1-5 mm) and *NS* No sensitive.

#### Effect of clove oil

Results in Table 6 show that 12 isolates were sensitive to clove oil. Three isolates were highly sensitive and identified as *A. niger* isolated from sample no. 11*, Rhizopus stolonifer* isolated from sample no. 11, and *Penicillium glabrum* isolated from sample no. 6, which represented clear zones of 10, 19, and 6 mm, respectively. Nine isolates showed moderate sensitivity to enzyme production when clove oil was used, which ranged from 1 to 5 mm. Clove oil was more effective against fungal isolates compared with the other peppermint and olive oils.

#### Effect of peppermint oil

The results in Table 6 show that one isolate was moderately sensitive which, identified as *Penicillium glabrum* and isolated from sample no. 3 furthermore, it showed a 3 mm clear zone, and the rest of the isolates showed no sensitivity.

#### Effect of olive oil

The results in Table 6 represent the moderately sensitive two isolates for olive oil, which were identified as *Rhizopus stolonifer* isolated from sample no. 11, and *Penicillium glabrum* isolated from sample no.11, The clear zone were 4 and 2 mm, respectively. The rest of the isolates showed no sensitivity.

## Discussion

Among the most hazardous microorganisms that can contaminate food and threaten the safety and quality of food, food products, and feed are fungi [33]. Up to 30% of crop products can be destroyed by fungus plant diseases, and rotting fungi and their toxins contaminate roughly 25% of the raw materials used in agriculture worldwide [34]. In Western Europe, the yearly economic damage resulting from fungi causing bread to decay is expected to exceed 200 million euros [35]. Foodborne diseases, often known as foodborne infections or food poisoning, represent a broad category of diseases caused by consuming harmful substances, pathogenic organisms, or spoiled food. Foodborne illnesses are a major contributor to morbidity and mortality, which makes them a significant worldwide impediment to socioeconomic growth [36]. There are many distinct types of foodborne diseases because food can be contaminated by a large range of harmful microbes.

According to estimates from the Centers for Disease Control and Prevention (CDC), 48 million Americans get sick from foodborne illnesses annually, 128,000 are admitted to hospitals, and 3000 pass away. Unsafe food, according to the WHO, results in 420,000 fatalities and 600 million cases of foodborne illnesses globally each year, with children under the age of five accounting for 30% of these cases. The World Health Organization calculated that eating contaminated food results in 33 million years of life loss annually worldwide [37]. The intrinsic characteristics of cakes, such as their pH level (5.2–8.7) and aw (0.78–0.96), make them susceptible to microbial deterioration [38]. Depending on the ingredients and product, filamentous mold, yeasts, and bacteria can spoil cakes. Elevated water activity levels could potentially encourage the growth of bacteria, leading to the spoiling of rope caused by Bacillus [39]. But since *Aspergillus*, *Penicillium*, and *Eurotium* are commonly identified in bread items, filamentous mold is recognized as the main suspect in food spoilage [7].

According to Garcia and Copetti [40], mold contamination of bread and bakery items is a problem that results in financial losses and dissatisfied customers. The growth of fungus on bakery items can cause contamination or product degradation. They have an impact on the product's quality. The product exhibits discoloration, has a lower capacity for storage or packing, and experiences certain chemical and physical changes. Fungus spoilage is another possible source of mycotoxins that might be harmful to the general public's health. Therefore, it is crucial to prevent fungal spoilage in bakery goods, and negative impacts can be reduced by using integrated strategies [41]. In the course of this inquiry, tainted baked goods were gathered. The quantity of these fungi grows daily as a result of the favorable storage conditions for these products, which is extremely dangerous because fungi are important food spoilage microorganisms that delay the nutritional value of food and occasionally produce mycotoxins, making it unfit for human consumption. The most significant and hazardous effect that fungi cause on human health is the production and release of mycotoxins in food [42].

Mycotoxicosis is a condition that can be either acute or chronic [43] and is caused by exposure to mycotoxins, such as ergotism, alimentary toxic aleukia, and aflatoxicosis. It affects various human organs and has the potential to be fatal. Mycotoxins can have a variety of harmful effects on both humans and animals after direct exposure, including endocrine abnormalities, carcinogenic, teratogenic, mutagenic, hemorrhagic, estrogenic, hepatotoxic, nephrotoxic, and immunosuppressive effects [11]. Mycotoxins are mostly introduced into the human body through contact, ingestion, and inhalation [44].

Two strategies are used to control mycotoxin contamination: detoxification and prevention of their formation [11]. Certain mycotoxins cannot be eliminated by conventional cooking methods. Mycotoxins can be removed from food in part or in whole utilizing a variety of food processing techniques as well as physical, chemical, and biological techniques [45]. The majority of mycotoxins maintain their chemical and thermal stability during various food processing techniques, such as boiling, baking, frying, baking, and pasteurizing. Mycotoxins, which are present in animal meals including meat, eggs, and milk as a result of contaminated feed, contaminate human food [46]. Because storage conditions have an effect on the general proliferation of fungi, they are crucial in controlling mycotoxins. Specifically, two primary elements—high humidity and temperature—can encourage the growth of fungi as well as the synthesis of mycotoxins. Mycotoxin accumulation and fungal growth are inhibited by storage in regulated settings, which include proper packing, temperature control, ventilation, and air humidity levels [47]. Inadequate storage techniques have been linked to crop losses of 20% to 50% in underdeveloped nations [48].

There are numerous methods for finding mycotoxins in food and food-related products. On the other hand, the most popular method is liquid chromatography coupled with tandem mass spectrometry (LC–MS/MS). With sufficient accuracy and precision, it can be utilized to detect various mycotoxins as well as masked or modified mycotoxins [49]. According to the study by Adeyeye [50], more fungal and mycotoxin contamination occurs in developing and tropical countries due to favorable environmental conditions than in industrialized and temperate countries [50]. Numerous variables, including temperature, humidity, pH, water activity (aw), nutrients, level of inoculation, substrate type, physiological condition, and microbial interactions, influence the growth and synthesis of mycotoxins in a variety of fungal species. Because of this, it is challenging for anyone to define the collection of ideal circumstances for development and output in physiological settings [51]. The normal conditions for fungal development are 10–40 ◦C, pH 8.4, and aw at values above 0.70 [51]. As well, the study by Kumari et al. [52] revealed that mold-induced microbiological deterioration is an issue for high-moisture items. This investigation supports our findings, which indicated that cakes containing water during manufacturing were contaminated by fungi.

So, fungus development on cakes is a regular issue. To establish practical controls to prevent post-baking contamination and product spoilage, knowledge of the fungus found in the manufacturing chain is required. These data are much more relevant in the current clean label environment [53]. Although several studies have sought to clarify the reason for the microbial deterioration of bakery products [23], little is known about their microbial biodiversity. In this study, the frequency and abundance of fungi in the chain of cake preparation were identified. According to El-Kadi et al. [54], *Penicillium* and *Aspergillus* species are frequent contaminants of cake, as in this investigation. Through medical research, it is known that these fungi are either toxin-producing or dangerous to humans and animals. These results are also agreement with Roussos et al. [55], who found that *Aspergillus* and *Penicillium* were the two major genera among mesophilic fungi, on both olives and olive cake sampled in Morocco.

In this research, cake samples were not contaminated with aflatoxin B1, B2, G1, G2, and ochratoxin, and this result correlated with the findings of Zain, who reported that some mold species can produce multiple mycotoxins; nevertheless, toxic fungal development is not always accompanied by mycotoxin production [56]. Furthermore, our study has similarities with the study of Sadaf et al. [56], who reported that out of 60 samples, 19 (32.0%) showed positive results for aflatoxin contamination, including 10% biscuits, 9% cakes, and 13% noodles, while 68% showed negative results for aflatoxins, also in agreement with [55, 57] who showed positive result for toxin production [55, 57], Interestingly, fungi isolated from cakes, such as *A. flavus* and *A. niger*, are known to produce mycotoxins, according to [55, 57], but aflatoxin B1, B2, G1, G2, and ochratoxin are not detected within the cake samples, and this may be due to Various factors affect the production of mycotoxins in many types of fungi, including temperature, humidity, environment, pH, water activity (aw), nutrients, level of inoculation, nature of the substrate, physiological state, microbial interactions [51], and the presence of preservatives in accurate doses.

Our results reported that most cake samples are contaminated with several fungal species, except cake samples no. 5 and 13 related to Funday company, showed no contamination with fungi, and this may be applied to all contents of cake samples within allowed limits, especially preservatives with perimitted ratios (potassium sorbate). We confirm our results from previous reports [52, 58], where, they reported that potassium sorbate was found to be the most effective in preventing fungal spoilage of this kind of product at the maximum concentration tested (0.3%), regardless of water activity.

Consumers and food producers do not like the widespread use of synthetic preservatives in the food industry since they are non-biodegradable, pose serious risks to human and animal health, and control mycotoxins in food items [59]. While mold species are becoming resistant to synthetic preservatives, their negative effects, such as the production of carcinogenic nitrosamines in food, are widely recognized [60]. As customers' knowledge of health issues has grown, the usage of chemical preservatives has been gradually curtailed. Food products can be biopreserved by natural or biological substances, which can help lower the incidence of foodborne illnesses and offer a good substitute for managing the microbiological deterioration of food and food products and the ensuing financial loss [61].

Essential oils are gaining popularity in comparison to other synthetic preservatives because they follow the current development trend of "green," "safety," and "health" food additives. Consequently, we began this research by providing an overview of the primary variables influencing the fungal contamination of baked goods. Afterward, the essential oil's antifungal activity and mechanism were examined. Essential oils are natural composites that are both volatile and non-volatile and include a complex mixture of terpenoids produced by aromatic plants as secondary metabolites. Traditional uses include natural flavorings, and more recently, natural antimicrobials for the preservation of food [62].

Since ancient times, clove, mint, and cinnamon have been used as food preservatives and medicinal herbs, primarily for their antioxidant and antibacterial properties.Recent investigations confirm that spice plants have antibacterial, antifungal, antiviral, and anti-carcinogenic capabilities. Particularly, clove possesses antibacterial and antioxidant properties [63]. Mandarin essential oil (EO) contains antibacterial and antioxidant properties [64, 65]. Essential oils from higher and aromatic plants have the ability to suppress the growth of microorganisms because essential oils contain secondary metabolites. EOs and its constituents have long been used by people worldwide to treat a variety of microbial diseases linked to the skin, fever, gastrointestinal tract, and respiratory system [66]. Strong antibacterial and antifungal effects have been demonstrated by clove, rosemary, and lavender oils.

The antibacterial properties of clove oil are demonstrated against a variety of harmful pathogens. As a cutting-edge alternative, clove oil microcapsules have practical applications as a preservative in meat products, especially in foods requiring heat processing. They had a strong heat resistance and high inhibition effect on meat products, and their disease indices and variation rates of mold spores also decreased [67].

In our results, three oils, including clove oil, peppermint oil, and olive oil, have been shown to have antifungal activity against various fungal strains with different values, in agreement with the results of the study [68] that found the physicochemical and thermal characteristics of nanocapsules were not significantly impacted by cinnamon or clove essential oils. While the other oils have a weak effect on the development of other fungi and a moderate effect on other fungi, they significantly increased the total phenolic content, antioxidant activity, antioxidant capacity, antifungal activity, and oxidative stability. And this result is consistent with the research findings of Malathy et al. [69], who reported that clove essential oil is a very effective, long-lasting, and secure natural substitute for chemical inhibitors for extending pita bread's shelf life. Future studies are necessary to establish the best techniques for encapsulating essential oils, as well as their solubility, absorption, bioavailability, shelf life, and impact on the organoleptic properties of food products.

The capacity of the fungal isolates to create the enzymes lipase, protease, cellulose, and amylase shows that they can biodegrade substrate-containing enzymes. [70], Fungi are mostly used in industry for the manufacturing of fermentation products, such as antibiotic enzymes and various biochemical compounds. Among the primary metabolites that fungi create are, ethanol, citric acid, gluconic acid, itaconic acid, amino acids, vitamins, nucleotides, and polysaccharides. Important secondary metabolites include antibiotics such as griseofulvin, fusidic acid, cephalosprins, and penicillin [71]. In this study, the extracellular enzymes Amylase, Protease, and Lipase produced by the selected fungi can help to break down complex carbohydrates, proteins, and lipids in solid substrates to simpler sugars, amino acids, and free fatty acids, thus affecting product quality as shown by other studies Orwa [72], Unachukwu & Nwakanma [73], and Garba et al. [70], revealed that three isolates from bread were isolated from contaminated sites, namely *Aspergillus* spp., *Penicillum* spp., and *Rhizopus* spp., and discovered that all isolates were positive for amylase and hence hydrolyzed. All isolates produced proteases, which is why skim milk was hydrolyzed. One was negative for lipase/esterase production while the rest two were positive, and these results are similar to our finding where most of the isolated fungi in our study, represented by *Aspergillus* spp., *Penicillum* spp., showed positive for amylase, protease, and lipase production for breaking down carbohydrates, lipids, and proteins in a cake sample to simple sugar.

Our results showed that most cake samples were contaminated with several fungal species, as well as cake samples no. 5 and 13 related to Funday company, which showed no contamination with fungi. This is because all contents of cake samples were within allowed limits, such as preservatives with permitted ratios (potassium sorbate).

The extracellular enzymes amylase, protease, and lipase produced by fungi break down complex carbohydrates, proteins, and lipids in solid substrates in to simpler sugars, amino acids, and free fatty acids, thus affecting product quality [16].

It is likely to be excreted when consumed, so the FAO recommends care and accuracy in manufacturing in food companies in order to prevent the growth of fungi because they produce toxins inside the consumer, in order to preserve public health. So the World Health Organization recommends paying careful attention to manufacturing in order to preserve the consumer. It was clear that the Funday company took care of the degree of absence of fungus by adding potassium sorbate as a preservative, so we thank those in charge of it, and we recommend that all companies take care of them. We also recommend adding clove, olive, and peppermint oil as antifungal agents during manufacturing to prevent microbial growth as a nature preservative. Therefore, in this paper, we first summarized the main factors affecting the fungal contamination of baked food. Then we analyzed the antifungal activity and mechanism of essential oil. Finally, we are promoting the application of essential oils in the preservation of baked food.

## Conclusion

According to the findings of this investigation, market cake samples appeared to be contaminated with fungi. The intrinsic characteristics of cakes, such as their pH level (5.2–8.7) and aw (0.78–0.96), make them susceptible to microbial deterioration. The study identified a diverse range of fungal species present in the market cake samples, predominantly including species from *Aspergillus* and *Penicillium*. The ability of the fungal isolates to produce amylase, protease, and lipase enzymes, demonstrate their ability to biodegrade enzymes containing substrate. Aflatoxins B_1_, B_2_, G_1_, G_2_ and ochratoxin A were not detected from fourteen collected samples of cake; however, toxic fungal development is not always accompanied by mycotoxin production: because storage conditions have an effect on the general proliferation of fungi and are crucial in controlling mycotoxins. Mycotoxin accumulation and fungal growth are inhibited by storage in regulated settings, which include proper packing, temperature control, and air humidity levels. Essential oils from higher and aromatic plants have the ability to suppress the growth of microorganisms because essential oils contain secondary metabolites, In this study, filamentous fungi isolated from cake samples were examined for their sensitivity to clove, peppermint, and olive oil. Clove oil was the better choice than peppermint oil and olive oil to prevent mold contamination. so the use of natural agents (EOs) is advantageous to preserving human health because chemical preservatives are harmful to human health. Food products can be biopreserved by natural or biological substances, which can help lower the incidence of foodborne illnesses and offer a good substitute for managing the microbiological deterioration of food and food products and the ensuing financial loss. Potassium sorbate was found to be the most effective in preventing fungal spoilage in bakery products.

## Data Availability

All data generated or analysed during this study are included in this published article."d.
